# 4-(5,6-Dihydro­benzimidazo[1,2-*c*]quinazolin-6-yl)benzene-1,3-diol dimethyl sulfoxide monosolvate

**DOI:** 10.1107/S160053681102304X

**Published:** 2011-06-18

**Authors:** Naser Eltaher Eltayeb, Siang Guan Teoh, Chin Sing Yeap, Hoong-Kun Fun

**Affiliations:** a School of Chemical Sciences, Universiti Sains Malaysia, 11800 USM, Penang, Malaysia; b Department of Chemistry, Faculty of Pure and Applied Sciences, International University of Africa, Sudan; c X-ray Crystallography Unit, School of Physics, Universiti Sains Malaysia, 11800 USM, Penang, Malaysia

## Abstract

In the title solvated benzimidazole compound, C_20_H_15_N_3_O_2_·C_2_H_6_OS, both the benzimidazole fused-ring system and the complete dimethyl sulfoxide solvent mol­ecule are disordered over two sets of sites, in 0.750 (5):0.250 (5) and 0.882 (4):0.118 (4) ratios, respectively. The conformation of the pyrimidine ring is close to a half-chair for the major disorder component, whereas for the minor component it is close to a boat. The dihy­droxy­phenyl ring is almost perpendicular to the mean plane of the benzimidazole ring [dihedral angle = 87.3 (2)° for the major disorder component and 88.3 (5)° for the minor disorder component]. In the crystal, mol­ecules are linked into layers parallel to (110) by O—H⋯N and C—H⋯O hydrogen bonds. A bifurcated O—H⋯(O,S) bond links the benzimidazole and solvent mol­ecules.

## Related literature

For related structures and background to benzimidazoles, see: Eltayeb *et al.* (2007*a* ▶,*b* ▶,*c* ▶, 2009 ▶). For the stability of the temperature controller used in the data collection, see: Cosier & Glazer (1986 ▶). For ring conformations, see: Cremer & Pople (1975 ▶).
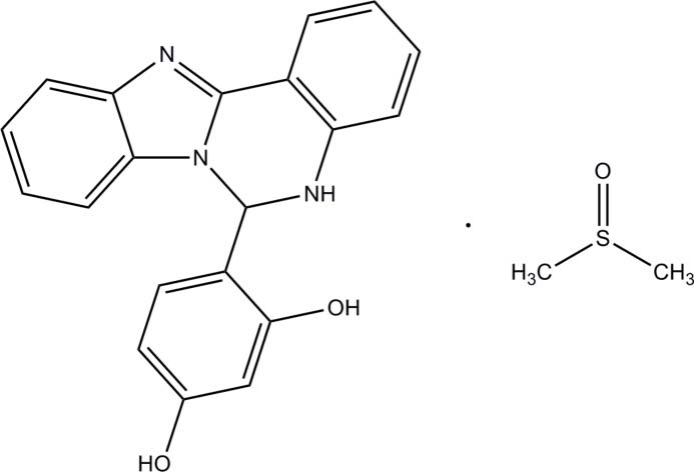


## Experimental

### 

#### Crystal data

C_20_H_15_N_3_O_2_·C_2_H_6_OS
*M* *_r_* = 407.48
Orthorhombic, 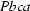

*a* = 9.9310 (18) Å
*b* = 16.342 (3) Å
*c* = 23.516 (5) Å
*V* = 3816.5 (13) Å^3^
*Z* = 8
Mo *K*α radiation
μ = 0.20 mm^−1^
*T* = 100 K
0.33 × 0.28 × 0.27 mm

#### Data collection

Bruker APEXII DUO CCD diffractometer
Absorption correction: multi-scan (*SADABS*; Bruker, 2009 ▶) *T*_min_ = 0.937, *T*_max_ = 0.949
22793 measured reflections
3345 independent reflections
3118 reflections with *I* > 2σ(*I*)
*R*_int_ = 0.031

#### Refinement

*R*[*F* ^2^ > 2σ(*F* ^2^)] = 0.096
*wR*(*F* ^2^) = 0.208
*S* = 1.28
3345 reflections
348 parameters
514 restraints
H-atom parameters constrained
Δρ_max_ = 0.33 e Å^−3^
Δρ_min_ = −0.37 e Å^−3^

### 

Data collection: *APEX2* (Bruker, 2009 ▶); cell refinement: *SAINT* (Bruker, 2009 ▶); data reduction: *SAINT*; program(s) used to solve structure: *SHELXTL* (Sheldrick, 2008 ▶); program(s) used to refine structure: *SHELXTL*; molecular graphics: *SHELXTL*; software used to prepare material for publication: *SHELXTL* and *PLATON* (Spek, 2009 ▶).

## Supplementary Material

Crystal structure: contains datablock(s) global, I. DOI: 10.1107/S160053681102304X/hb5886sup1.cif

Structure factors: contains datablock(s) I. DOI: 10.1107/S160053681102304X/hb5886Isup2.hkl

Supplementary material file. DOI: 10.1107/S160053681102304X/hb5886Isup3.cml

Additional supplementary materials:  crystallographic information; 3D view; checkCIF report

## Figures and Tables

**Table 1:** Hydrogen-bond geometry (Å, °)

*D*—H⋯*A*	*D*—H	H⋯*A*	*D*⋯*A*	*D*—H⋯*A*
O1—H1*O*1⋯S1*A*	0.94	2.82	3.732 (3)	163
O1—H1*O*1⋯O3*A*	0.94	1.71	2.619 (9)	163
O2—H1*O*2⋯N2*A*^i^	0.88	1.95	2.739 (6)	150
C11*A*—H11*A*⋯O2^ii^	0.93	2.40	3.329 (9)	174

Symmetry codes: (i) 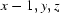; (ii) 
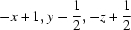
.

**Fig. 1.**
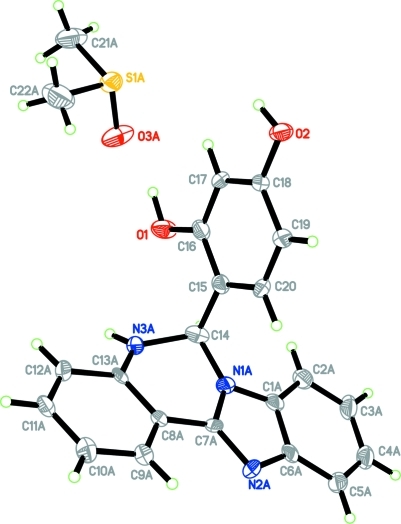
The molecular structure of (I) with 30% probability ellipsoids for non-H atoms. Only major disordered component is shown.

**Fig. 2.**
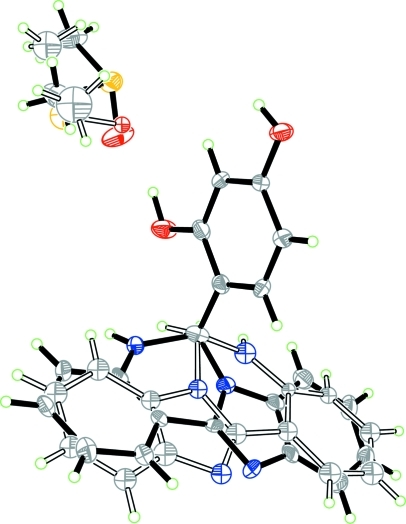
The molecular structure of (I), showing all disordered components.

**Fig. 3.**
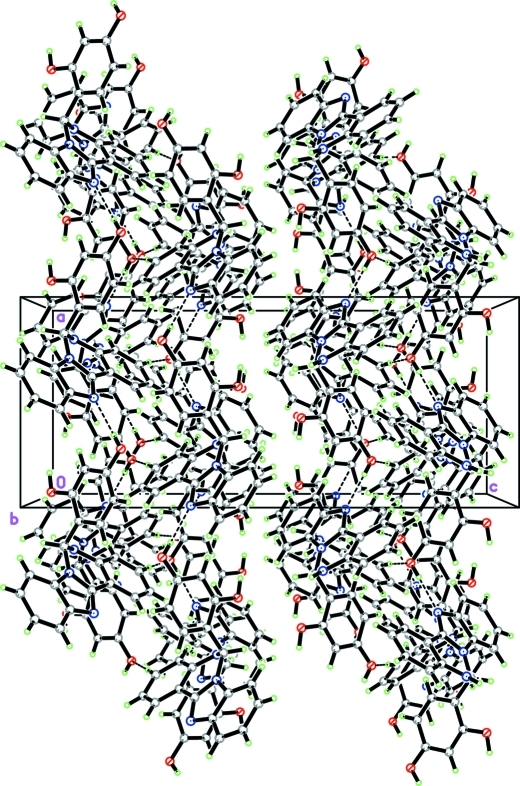
The packing of (I), viewed down b axis, showing molecules are linked into plane parallel to ab plane. Only major disordered component is shown. Solvent molecules are omitted for clarity and hydrogen bonds are shown as dashed lines.

## supplementary crystallographic information

### Comment

As part of our ongoing structural studies of benzimidazoles (Eltayeb *et al.*, 2007**a*,*b*,c*, 2009) we now describe in this paper the single-crystal X-ray diffraction study of title compound, (I), (Fig. 1). Furthermore, this paper describes for the first time a simple method for synthesis of benzimidazo[1,2-*c*]quinazoline derivatives using zinc chloride as a homogenous catalyst, herein, and thereafter to be called the "Taha-Teoh's method".

The benzimidazole fused ring system in (I) is disordered over two sets of sites with refined site occupancies of 0.750 (5) and 0.250 (5). The solvent molecule is also disordered over two orientations with refined site occupancies of 0.882 (4) and 0.118 (4). The conformation for pyrimidine ring is close to a half-chair conformation for major component whereas for minor component it is close to a boat conformation (Cremer & Pople, 1975). The dihydroxyphenyl ring is almost perpendicular to the mean plane of benzimidazole ring (N1A/C1A–C6A/N2A/C7A) with the dihedral angle of 87.3 (2)° whereas this angle is 88.3 (5)° for minor component. In the crystal structure, the molecules are linked into infinite one-dimensional chains along *a* axis by intermolecular O2—H1O2···N2A hydrogen bonds and the intermolecular C11A—H11A···O2 hydrogen bonds (Table 1) further linked these chains into planes parallel to *ab* plane (Fig. 3). The benzimidazole molecule and the solvent molecule is stabilized by the O1—H1O1···S1A and O1—H1O1···O3A interactions (Table 1).

### Experimental

To a solution of 2-(2-aminophenyl)-1*H*-benzimidazole (0.209 g, 1.0 mmol) in ethanol (30 ml) was added 2,4-dihydroxybenzaldehyde (0.138 g, 1.0 mmol). The color of the resulting solution is pale-pink. Then on adding zinc chloride (0.136 g, 1.0 mmol), the color of solution changed to yellowish-pink. The mixture was refluxed with stirring for 3 hours. The product (yellow precipitate) was obtained by evaporation of the solvent under reduced pressure using a rotary evaporator.  Yellow blocks of (I) were formed after several days of slow evaporation of an acetone solution layered with a small amount of dimethylsulfoxide at room temperature.

### Refinement

All disordered components were subjected to rigid bond and similarity restraints. All minor disordered components were refined isotropically. The O-bound hydrogen atoms were located from difference Fourier map and refined as riding on their parent atom, with *U*_iso_(H) = 1.5 *U*_eq_(O). The rest of the hydrogen atoms were positioned geomatrically [C–H = 0.93–0.98 Å; N–H = 0.86 Å] and refined using a riding model, with *U*_iso_(H) = 1.2 or 1.5 *U*_eq_(C,*N* or C_methyl_). A rotating-group model were applied for methyl groups.

### Crystal data

**Table:**

C_20_H_15_N_3_O_2_·C_2_H_6_OS	*F*(000) = 1712
*M**_r_* = 407.48	*D*_x_ = 1.418 Mg m^−^^3^
Orthorhombic, *P**b**c**a*	Mo *K*α radiation, λ = 0.71073 Å
Hall symbol: -P 2ac 2ab	Cell parameters from 9998 reflections
*a* = 9.9310 (18) Å	θ = 2.5–29.9°
*b* = 16.342 (3) Å	µ = 0.20 mm^−^^1^
*c* = 23.516 (5) Å	*T* = 100 K
*V* = 3816.5 (13) Å^3^	Block, yellow
*Z* = 8	0.33 × 0.28 × 0.27 mm

### Data collection

**Table:**

Bruker APEXII DUO CCD diffractometer	3345 independent reflections
Radiation source: fine-focus sealed tube	3118 reflections with *I* > 2σ(*I*)
graphite	*R*_int_ = 0.031
φ and ω scans	θ_max_ = 25.0°, θ_min_ = 2.6°
Absorption correction: multi-scan (*SADABS*; Bruker, 2009)	*h* = −11→11
*T*_min_ = 0.937, *T*_max_ = 0.949	*k* = −19→19
22793 measured reflections	*l* = −27→27

### Refinement

**Table:**

Refinement on *F*^2^	Primary atom site location: structure-invariant direct methods
Least-squares matrix: full	Secondary atom site location: difference Fourier map
*R*[*F*^2^ > 2σ(*F*^2^)] = 0.096	Hydrogen site location: inferred from neighbouring sites
*wR*(*F*^2^) = 0.208	H-atom parameters constrained
*S* = 1.28	*w* = 1/[σ^2^(*F*_o_^2^) + (0.*P*)^2^ + 23.9034*P*] where *P* = (*F*_o_^2^ + 2*F*_c_^2^)/3
3345 reflections	(Δ/σ)_max_ < 0.001
348 parameters	Δρ_max_ = 0.33 e Å^−^^3^
514 restraints	Δρ_min_ = −0.37 e Å^−^^3^

### Special details

**Table:**

Experimental. The crystal was placed in the cold stream of an Oxford Cryosystems Cobra open-flow nitrogen cryostat (Cosier & Glazer, 1986) operating at 100.0 (1) K.
Geometry. All e.s.d.'s (except the e.s.d. in the dihedral angle between two l.s. planes) are estimated using the full covariance matrix. The cell e.s.d.'s are taken into account individually in the estimation of e.s.d.'s in distances, angles and torsion angles; correlations between e.s.d.'s in cell parameters are only used when they are defined by crystal symmetry. An approximate (isotropic) treatment of cell e.s.d.'s is used for estimating e.s.d.'s involving l.s. planes.
Refinement. Refinement of *F*^2^ against ALL reflections. The weighted *R*-factor *wR* and goodness of fit *S* are based on *F*^2^, conventional *R*-factors *R* are based on *F*, with *F* set to zero for negative *F*^2^. The threshold expression of *F*^2^ > σ(*F*^2^) is used only for calculating *R*-factors(gt) *etc*. and is not relevant to the choice of reflections for refinement. *R*-factors based on *F*^2^ are statistically about twice as large as those based on *F*, and *R*- factors based on ALL data will be even larger.

### Fractional atomic coordinates and isotropic or equivalent isotropic displacement parameters (Å^2^)

**Table:**

	*x*	*y*	*z*	*U*_iso_*/*U*_eq_	Occ. (<1)
O1	0.4247 (3)	0.5246 (2)	0.06384 (15)	0.0314 (8)	
H1O1	0.3349	0.5081	0.0588	0.047*	
O2	0.2150 (3)	0.6868 (2)	0.20967 (16)	0.0310 (8)	
H1O2	0.1510	0.6572	0.1944	0.047*	
N1A	0.7956 (5)	0.6144 (3)	0.1050 (2)	0.0221 (12)	0.750 (5)
N2A	0.9792 (5)	0.6498 (3)	0.1550 (2)	0.0242 (12)	0.750 (5)
N3A	0.7010 (5)	0.4845 (3)	0.1064 (2)	0.0234 (12)	0.750 (5)
H3AB	0.6761	0.4464	0.0834	0.028*	0.750 (5)
C1A	0.8411 (7)	0.6865 (4)	0.0810 (4)	0.0241 (15)	0.750 (5)
C2A	0.7922 (7)	0.7319 (5)	0.0355 (3)	0.0279 (15)	0.750 (5)
H2AA	0.7158	0.7164	0.0153	0.033*	0.750 (5)
C3A	0.8652 (8)	0.8019 (5)	0.0221 (3)	0.0327 (17)	0.750 (5)
H3AA	0.8355	0.8353	−0.0074	0.039*	0.750 (5)
C4A	0.9809 (9)	0.8230 (4)	0.0515 (4)	0.0324 (17)	0.750 (5)
H4AA	1.0281	0.8696	0.0406	0.039*	0.750 (5)
C5A	1.0286 (7)	0.7770 (4)	0.0966 (4)	0.0288 (16)	0.750 (5)
H5AA	1.1064	0.7918	0.1160	0.035*	0.750 (5)
C6A	0.9545 (8)	0.7069 (4)	0.1120 (3)	0.0226 (14)	0.750 (5)
C7A	0.8802 (6)	0.5969 (4)	0.1489 (3)	0.0190 (13)	0.750 (5)
C8A	0.8574 (8)	0.5213 (4)	0.1808 (3)	0.0203 (15)	0.750 (5)
C9A	0.9260 (7)	0.5022 (4)	0.2303 (3)	0.0269 (14)	0.750 (5)
H9AA	0.9850	0.5403	0.2458	0.032*	0.750 (5)
C10A	0.9087 (10)	0.4281 (5)	0.2569 (4)	0.030 (2)	0.750 (5)
H10A	0.9562	0.4156	0.2898	0.036*	0.750 (5)
C11A	0.8185 (8)	0.3722 (5)	0.2334 (4)	0.0282 (19)	0.750 (5)
H11A	0.8052	0.3220	0.2512	0.034*	0.750 (5)
C12A	0.7486 (6)	0.3901 (4)	0.1842 (3)	0.0269 (15)	0.750 (5)
H12A	0.6903	0.3515	0.1688	0.032*	0.750 (5)
C13A	0.7646 (6)	0.4660 (4)	0.1571 (3)	0.0209 (13)	0.750 (5)
N1B	0.7996 (14)	0.5740 (8)	0.1323 (6)	0.019 (3)*	0.250 (5)
N2B	0.9819 (14)	0.6061 (8)	0.1829 (6)	0.021 (3)*	0.250 (5)
N3B	0.7269 (15)	0.6445 (9)	0.0489 (7)	0.027 (4)*	0.250 (5)
H3BB	0.6960	0.6426	0.0148	0.032*	0.250 (5)
C1B	0.818 (2)	0.5089 (12)	0.1694 (9)	0.019 (5)*	0.250 (5)
C2B	0.747 (2)	0.4368 (13)	0.1770 (9)	0.025 (5)*	0.250 (5)
H2BA	0.6716	0.4247	0.1551	0.030*	0.250 (5)
C3B	0.794 (3)	0.3833 (15)	0.2191 (12)	0.026 (7)*	0.250 (5)
H3BA	0.7516	0.3332	0.2251	0.031*	0.250 (5)
C4B	0.903 (3)	0.4067 (14)	0.2515 (13)	0.022 (7)*	0.250 (5)
H4BA	0.9305	0.3717	0.2805	0.027*	0.250 (5)
C5B	0.976 (2)	0.4780 (11)	0.2440 (8)	0.023 (5)*	0.250 (5)
H5BA	1.0494	0.4911	0.2667	0.028*	0.250 (5)
C6B	0.932 (2)	0.5296 (10)	0.1998 (8)	0.029 (5)*	0.250 (5)
C7B	0.902 (2)	0.6279 (10)	0.1412 (8)	0.024 (5)*	0.250 (5)
C8B	0.904 (2)	0.7028 (12)	0.1076 (9)	0.022 (6)*	0.250 (5)
C9B	1.001 (2)	0.7629 (13)	0.1161 (9)	0.027 (6)*	0.250 (5)
H9BA	1.0598	0.7584	0.1468	0.032*	0.250 (5)
C10B	1.012 (2)	0.8281 (13)	0.0804 (9)	0.030 (5)*	0.250 (5)
H10B	1.0761	0.8686	0.0866	0.036*	0.250 (5)
C11B	0.925 (2)	0.8325 (13)	0.0348 (9)	0.025 (5)*	0.250 (5)
H11B	0.9264	0.8788	0.0118	0.029*	0.250 (5)
C12B	0.835 (2)	0.7708 (13)	0.0221 (8)	0.017 (5)*	0.250 (5)
H12B	0.7886	0.7710	−0.0123	0.020*	0.250 (5)
C13B	0.8146 (19)	0.7077 (11)	0.0616 (8)	0.011 (4)*	0.250 (5)
C14	0.6765 (5)	0.5734 (3)	0.0925 (2)	0.0274 (11)	
H14A	0.6600	0.5780	0.0516	0.033*	0.750 (5)
H14B	0.6588	0.5220	0.0743	0.033*	0.250 (5)
C15	0.5554 (5)	0.6069 (3)	0.1234 (2)	0.0215 (10)	
C16	0.4288 (5)	0.5788 (3)	0.1079 (2)	0.0230 (10)	
C17	0.3134 (4)	0.6063 (3)	0.1349 (2)	0.0208 (10)	
H17A	0.2291	0.5887	0.1228	0.025*	
C18	0.3246 (4)	0.6602 (3)	0.1801 (2)	0.0220 (10)	
C19	0.4505 (4)	0.6897 (3)	0.1959 (2)	0.0239 (10)	
H19A	0.4583	0.7274	0.2253	0.029*	
C20	0.5633 (5)	0.6628 (3)	0.1677 (2)	0.0223 (10)	
H20A	0.6472	0.6826	0.1786	0.027*	
S1A	0.07237 (15)	0.44769 (9)	0.07749 (7)	0.0329 (5)	0.882 (4)
O3A	0.1950 (8)	0.4548 (5)	0.0386 (4)	0.045 (2)	0.882 (4)
C21A	−0.0447 (6)	0.3857 (5)	0.0400 (4)	0.0478 (19)	0.882 (4)
H21A	−0.0817	0.4163	0.0088	0.072*	0.882 (4)
H21B	−0.1160	0.3695	0.0652	0.072*	0.882 (4)
H21C	0.0002	0.3379	0.0257	0.072*	0.882 (4)
C22A	0.1182 (9)	0.3767 (6)	0.1298 (4)	0.056 (2)	0.882 (4)
H22A	0.1978	0.3954	0.1490	0.083*	0.882 (4)
H22B	0.1357	0.3247	0.1124	0.083*	0.882 (4)
H22C	0.0462	0.3711	0.1568	0.083*	0.882 (4)
S1B	0.1514 (13)	0.3778 (8)	0.0710 (6)	0.045 (4)*	0.118 (4)
O3B	0.168 (5)	0.461 (2)	0.042 (2)	0.015 (10)*	0.118 (4)
C21B	−0.020 (3)	0.352 (3)	0.060 (2)	0.049 (16)*	0.118 (4)
H21D	−0.0723	0.4010	0.0553	0.074*	0.118 (4)
H21E	−0.0533	0.3215	0.0916	0.074*	0.118 (4)
H21F	−0.0280	0.3193	0.0259	0.074*	0.118 (4)
C22B	0.140 (8)	0.400 (5)	0.1440 (10)	0.07 (3)*	0.118 (4)
H22D	0.2266	0.4179	0.1576	0.104*	0.118 (4)
H22E	0.1125	0.3523	0.1643	0.104*	0.118 (4)
H22F	0.0755	0.4432	0.1497	0.104*	0.118 (4)

### Atomic displacement parameters (Å^2^)

**Table:**

	*U*^11^	*U*^22^	*U*^33^	*U*^12^	*U*^13^	*U*^23^
O1	0.0202 (17)	0.039 (2)	0.035 (2)	0.0028 (15)	−0.0027 (15)	−0.0165 (16)
O2	0.0164 (16)	0.0299 (18)	0.047 (2)	−0.0039 (14)	0.0039 (15)	−0.0156 (16)
N1A	0.016 (2)	0.021 (3)	0.030 (3)	0.000 (2)	0.003 (2)	0.004 (2)
N2A	0.017 (3)	0.018 (3)	0.037 (3)	−0.005 (2)	0.002 (2)	0.000 (2)
N3A	0.019 (3)	0.016 (2)	0.036 (3)	0.001 (2)	−0.001 (2)	−0.007 (2)
C1A	0.015 (3)	0.020 (3)	0.037 (4)	0.004 (3)	0.009 (3)	0.003 (3)
C2A	0.024 (3)	0.024 (4)	0.035 (4)	0.003 (3)	0.007 (3)	0.007 (3)
C3A	0.036 (4)	0.018 (4)	0.044 (4)	−0.002 (4)	0.011 (3)	0.002 (3)
C4A	0.032 (4)	0.017 (3)	0.048 (5)	−0.002 (3)	0.013 (4)	0.003 (3)
C5A	0.021 (3)	0.021 (4)	0.045 (4)	−0.003 (3)	0.012 (3)	0.000 (3)
C6A	0.019 (4)	0.017 (3)	0.032 (4)	0.006 (3)	0.009 (3)	−0.003 (2)
C7A	0.010 (3)	0.018 (3)	0.030 (3)	0.001 (2)	0.004 (2)	−0.004 (3)
C8A	0.013 (4)	0.015 (3)	0.033 (4)	0.004 (2)	0.002 (3)	−0.002 (3)
C9A	0.021 (3)	0.018 (3)	0.042 (4)	0.002 (3)	0.000 (3)	−0.001 (3)
C10A	0.032 (4)	0.022 (4)	0.036 (4)	0.003 (4)	−0.003 (3)	0.004 (4)
C11A	0.022 (4)	0.017 (3)	0.046 (5)	0.006 (3)	0.004 (4)	0.008 (3)
C12A	0.021 (3)	0.014 (3)	0.045 (4)	0.001 (2)	0.005 (3)	−0.001 (3)
C13A	0.013 (3)	0.014 (3)	0.036 (4)	0.007 (2)	0.006 (3)	−0.001 (3)
C14	0.018 (2)	0.033 (3)	0.031 (3)	0.006 (2)	−0.001 (2)	0.000 (2)
C15	0.022 (2)	0.018 (2)	0.025 (2)	0.0022 (18)	0.0027 (19)	0.0044 (18)
C16	0.024 (2)	0.018 (2)	0.027 (2)	0.0045 (19)	−0.004 (2)	−0.0021 (19)
C17	0.016 (2)	0.017 (2)	0.030 (3)	−0.0035 (17)	−0.0017 (19)	0.0008 (19)
C18	0.014 (2)	0.017 (2)	0.035 (3)	0.0005 (17)	0.0036 (19)	0.0002 (19)
C19	0.019 (2)	0.021 (2)	0.032 (3)	0.0000 (18)	−0.001 (2)	−0.005 (2)
C20	0.019 (2)	0.016 (2)	0.033 (3)	−0.0041 (18)	−0.003 (2)	0.0026 (19)
S1A	0.0251 (8)	0.0290 (8)	0.0447 (9)	−0.0031 (6)	0.0018 (7)	−0.0083 (7)
O3A	0.029 (4)	0.062 (4)	0.045 (3)	−0.017 (3)	0.007 (3)	−0.013 (3)
C21A	0.025 (3)	0.060 (5)	0.059 (5)	−0.008 (3)	0.004 (3)	−0.021 (4)
C22A	0.039 (4)	0.062 (6)	0.066 (5)	0.018 (4)	0.008 (4)	0.021 (5)

### Geometric parameters (Å, °)

**Table:**

O1—C16	1.363 (5)	C3B—H3BA	0.9300
O1—H1O1	0.9391	C4B—C5B	1.382 (19)
O2—C18	1.362 (5)	C4B—H4BA	0.9300
O2—H1O2	0.8748	C5B—C6B	1.408 (17)
N1A—C7A	1.362 (8)	C5B—H5BA	0.9300
N1A—C1A	1.381 (8)	C7B—C8B	1.457 (15)
N1A—C14	1.391 (7)	C8B—C9B	1.395 (18)
N2A—C7A	1.317 (7)	C8B—C13B	1.399 (17)
N2A—C6A	1.397 (8)	C9B—C10B	1.361 (18)
N3A—C13A	1.382 (8)	C9B—H9BA	0.9300
N3A—C14	1.509 (7)	C10B—C11B	1.381 (18)
N3A—H3AB	0.8600	C10B—H10B	0.9300
N3A—H14B	1.0594	C11B—C12B	1.378 (18)
C1A—C6A	1.383 (11)	C11B—H11B	0.9300
C1A—C2A	1.390 (10)	C12B—C13B	1.403 (17)
C2A—C3A	1.390 (10)	C12B—H12B	0.9300
C2A—H2AA	0.9300	C14—C15	1.508 (6)
C3A—C4A	1.386 (12)	C14—H14A	0.9800
C3A—H3AA	0.9300	C14—H14B	0.9601
C4A—C5A	1.384 (12)	C15—C16	1.387 (6)
C4A—H4AA	0.9300	C15—C20	1.388 (6)
C5A—C6A	1.408 (10)	C16—C17	1.386 (6)
C5A—H5AA	0.9300	C17—C18	1.384 (7)
C7A—C8A	1.463 (9)	C17—H17A	0.9300
C8A—C9A	1.383 (11)	C18—C19	1.391 (6)
C8A—C13A	1.406 (10)	C19—C20	1.373 (6)
C9A—C10A	1.373 (11)	C19—H19A	0.9300
C9A—H9AA	0.9300	C20—H20A	0.9300
C10A—C11A	1.393 (11)	S1A—O3A	1.528 (7)
C10A—H10A	0.9300	S1A—C22A	1.752 (8)
C11A—C12A	1.381 (11)	S1A—C21A	1.776 (6)
C11A—H11A	0.9300	C21A—H21A	0.9600
C12A—C13A	1.404 (9)	C21A—H21B	0.9600
C12A—H12A	0.9300	C21A—H21C	0.9600
N1B—C7B	1.359 (15)	C22A—H22A	0.9600
N1B—C1B	1.388 (16)	C22A—H22B	0.9600
N1B—C14	1.540 (14)	C22A—H22C	0.9600
N2B—C7B	1.313 (15)	S1B—O3B	1.534 (19)
N2B—C6B	1.401 (15)	S1B—C22B	1.76 (2)
N3B—C13B	1.384 (16)	S1B—C21B	1.776 (19)
N3B—C14	1.629 (15)	C21B—H21D	0.9600
N3B—H3BB	0.8600	C21B—H21E	0.9600
C1B—C6B	1.378 (17)	C21B—H21F	0.9600
C1B—C2B	1.387 (17)	C22B—H22D	0.9600
C2B—C3B	1.399 (18)	C22B—H22E	0.9600
C2B—H2BA	0.9300	C22B—H22F	0.9600
C3B—C4B	1.38 (2)		
			
C16—O1—H1O1	108.1	N2B—C7B—C8B	128.8 (15)
C18—O2—H1O2	101.3	N1B—C7B—C8B	118.0 (13)
C7A—N1A—C1A	106.7 (6)	C9B—C8B—C13B	120.6 (14)
C7A—N1A—C14	125.6 (5)	C9B—C8B—C7B	121.5 (16)
C1A—N1A—C14	127.1 (6)	C13B—C8B—C7B	117.4 (16)
C7A—N2A—C6A	103.3 (6)	C10B—C9B—C8B	121.2 (16)
C13A—N3A—C14	118.1 (5)	C10B—C9B—H9BA	119.4
C13A—N3A—H3AB	121.0	C8B—C9B—H9BA	119.4
C14—N3A—H3AB	121.0	C9B—C10B—C11B	118.1 (17)
C13A—N3A—H14B	157.2	C9B—C10B—H10B	120.9
H3AB—N3A—H14B	81.8	C11B—C10B—H10B	120.9
N1A—C1A—C6A	104.9 (7)	C12B—C11B—C10B	122.4 (17)
N1A—C1A—C2A	131.0 (7)	C12B—C11B—H11B	118.8
C6A—C1A—C2A	124.2 (6)	C10B—C11B—H11B	118.8
C3A—C2A—C1A	115.6 (6)	C11B—C12B—C13B	119.2 (15)
C3A—C2A—H2AA	122.2	C11B—C12B—H12B	120.4
C1A—C2A—H2AA	122.2	C13B—C12B—H12B	120.4
C4A—C3A—C2A	121.7 (7)	N3B—C13B—C8B	121.4 (17)
C4A—C3A—H3AA	119.2	N3B—C13B—C12B	119.8 (16)
C2A—C3A—H3AA	119.2	C8B—C13B—C12B	117.6 (13)
C5A—C4A—C3A	122.0 (7)	N1A—C14—C15	113.7 (4)
C5A—C4A—H4AA	119.0	N1A—C14—N3A	106.4 (4)
C3A—C4A—H4AA	119.0	C15—C14—N3A	111.9 (4)
C4A—C5A—C6A	117.4 (7)	C15—C14—N1B	109.8 (6)
C4A—C5A—H5AA	121.3	N3A—C14—N1B	75.3 (6)
C6A—C5A—H5AA	121.3	N1A—C14—N3B	61.8 (6)
C1A—C6A—N2A	111.3 (7)	C15—C14—N3B	106.8 (6)
C1A—C6A—C5A	119.1 (7)	N3A—C14—N3B	140.7 (7)
N2A—C6A—C5A	129.6 (8)	N1B—C14—N3B	97.7 (7)
N2A—C7A—N1A	113.9 (6)	N1A—C14—H14A	108.2
N2A—C7A—C8A	127.9 (6)	C15—C14—H14A	108.2
N1A—C7A—C8A	118.1 (6)	N3A—C14—H14A	108.2
C9A—C8A—C13A	120.7 (6)	N1B—C14—H14A	136.9
C9A—C8A—C7A	123.1 (7)	N3B—C14—H14A	51.5
C13A—C8A—C7A	116.1 (7)	N1A—C14—H14B	132.2
C10A—C9A—C8A	121.4 (7)	C15—C14—H14B	112.7
C10A—C9A—H9AA	119.3	N1B—C14—H14B	115.0
C8A—C9A—H9AA	119.3	N3B—C14—H14B	113.5
C9A—C10A—C11A	118.5 (8)	H14A—C14—H14B	66.2
C9A—C10A—H10A	120.7	C16—C15—C20	117.8 (4)
C11A—C10A—H10A	120.7	C16—C15—C14	118.4 (4)
C12A—C11A—C10A	121.1 (7)	C20—C15—C14	123.8 (4)
C12A—C11A—H11A	119.5	O1—C16—C17	122.3 (4)
C10A—C11A—H11A	119.5	O1—C16—C15	116.3 (4)
C11A—C12A—C13A	120.7 (6)	C17—C16—C15	121.4 (4)
C11A—C12A—H12A	119.6	C18—C17—C16	119.5 (4)
C13A—C12A—H12A	119.6	C18—C17—H17A	120.2
N3A—C13A—C12A	122.2 (6)	C16—C17—H17A	120.2
N3A—C13A—C8A	120.1 (7)	O2—C18—C17	122.0 (4)
C12A—C13A—C8A	117.5 (6)	O2—C18—C19	118.2 (4)
C7B—N1B—C1B	107.4 (13)	C17—C18—C19	119.8 (4)
C7B—N1B—C14	133.6 (11)	C20—C19—C18	119.6 (4)
C1B—N1B—C14	118.9 (12)	C20—C19—H19A	120.2
C7B—N2B—C6B	103.8 (12)	C18—C19—H19A	120.2
C13B—N3B—C14	126.2 (13)	C19—C20—C15	121.8 (4)
C13B—N3B—H3BB	116.9	C19—C20—H20A	119.1
C14—N3B—H3BB	116.9	C15—C20—H20A	119.1
C6B—C1B—C2B	124.2 (14)	O3A—S1A—C22A	105.3 (4)
C6B—C1B—N1B	104.3 (14)	O3A—S1A—C21A	105.5 (4)
C2B—C1B—N1B	131.5 (17)	C22A—S1A—C21A	98.1 (5)
C1B—C2B—C3B	116.9 (16)	O3B—S1B—C22B	105 (2)
C1B—C2B—H2BA	121.5	O3B—S1B—C21B	104.4 (19)
C3B—C2B—H2BA	121.5	C22B—S1B—C21B	97.8 (19)
C4B—C3B—C2B	118.5 (18)	S1B—C21B—H21D	109.5
C4B—C3B—H3BA	120.7	S1B—C21B—H21E	109.5
C2B—C3B—H3BA	120.7	H21D—C21B—H21E	109.5
C5B—C4B—C3B	125.0 (19)	S1B—C21B—H21F	109.5
C5B—C4B—H4BA	117.5	H21D—C21B—H21F	109.5
C3B—C4B—H4BA	117.5	H21E—C21B—H21F	109.5
C4B—C5B—C6B	116.0 (16)	S1B—C22B—H22D	109.5
C4B—C5B—H5BA	122.0	S1B—C22B—H22E	109.5
C6B—C5B—H5BA	122.0	H22D—C22B—H22E	109.5
C1B—C6B—N2B	111.2 (14)	S1B—C22B—H22F	109.5
C1B—C6B—C5B	119.3 (14)	H22D—C22B—H22F	109.5
N2B—C6B—C5B	129.3 (16)	H22E—C22B—H22F	109.5
N2B—C7B—N1B	113.0 (12)		
			
C7A—N1A—C1A—C6A	1.5 (7)	N2B—C7B—C8B—C9B	2(4)
C14—N1A—C1A—C6A	173.1 (5)	N1B—C7B—C8B—C9B	177 (2)
C7A—N1A—C1A—C2A	−179.6 (7)	N2B—C7B—C8B—C13B	174 (2)
C14—N1A—C1A—C2A	−8.0 (11)	N1B—C7B—C8B—C13B	−11 (4)
N1A—C1A—C2A—C3A	−179.0 (7)	C13B—C8B—C9B—C10B	0(4)
C6A—C1A—C2A—C3A	−0.4 (10)	C7B—C8B—C9B—C10B	172 (2)
C1A—C2A—C3A—C4A	1.8 (10)	C8B—C9B—C10B—C11B	−1(4)
C2A—C3A—C4A—C5A	−1.6 (11)	C9B—C10B—C11B—C12B	−5(4)
C3A—C4A—C5A—C6A	0.0 (10)	C10B—C11B—C12B—C13B	11 (4)
N1A—C1A—C6A—N2A	−0.8 (7)	C14—N3B—C13B—C8B	21 (3)
C2A—C1A—C6A—N2A	−179.8 (6)	C14—N3B—C13B—C12B	−171.4 (16)
N1A—C1A—C6A—C5A	177.7 (6)	C9B—C8B—C13B—N3B	173 (2)
C2A—C1A—C6A—C5A	−1.2 (10)	C7B—C8B—C13B—N3B	1(4)
C7A—N2A—C6A—C1A	−0.2 (7)	C9B—C8B—C13B—C12B	6(4)
C7A—N2A—C6A—C5A	−178.6 (7)	C7B—C8B—C13B—C12B	−167 (2)
C4A—C5A—C6A—C1A	1.4 (10)	C11B—C12B—C13B—N3B	−179 (2)
C4A—C5A—C6A—N2A	179.6 (7)	C11B—C12B—C13B—C8B	−11 (3)
C6A—N2A—C7A—N1A	1.2 (7)	C7A—N1A—C14—C15	87.5 (7)
C6A—N2A—C7A—C8A	176.9 (6)	C1A—N1A—C14—C15	−82.6 (7)
C1A—N1A—C7A—N2A	−1.8 (7)	C7A—N1A—C14—N3A	−36.1 (7)
C14—N1A—C7A—N2A	−173.6 (5)	C1A—N1A—C14—N3A	153.8 (6)
C1A—N1A—C7A—C8A	−177.9 (6)	C7A—N1A—C14—N1B	−4.0 (10)
C14—N1A—C7A—C8A	10.3 (9)	C1A—N1A—C14—N1B	−174.1 (12)
N2A—C7A—C8A—C9A	12.6 (11)	C7A—N1A—C14—N3B	−175.4 (9)
N1A—C7A—C8A—C9A	−171.9 (6)	C1A—N1A—C14—N3B	14.5 (8)
N2A—C7A—C8A—C13A	−164.7 (6)	C13A—N3A—C14—N1A	45.1 (6)
N1A—C7A—C8A—C13A	10.8 (9)	C13A—N3A—C14—C15	−79.6 (6)
C13A—C8A—C9A—C10A	1.8 (11)	C13A—N3A—C14—N1B	26.1 (7)
C7A—C8A—C9A—C10A	−175.4 (8)	C13A—N3A—C14—N3B	110.4 (11)
C8A—C9A—C10A—C11A	−0.9 (14)	C7B—N1B—C14—N1A	9.0 (16)
C9A—C10A—C11A—C12A	0.6 (14)	C1B—N1B—C14—N1A	−172 (2)
C10A—C11A—C12A—C13A	−1.3 (12)	C7B—N1B—C14—C15	−94 (2)
C14—N3A—C13A—C12A	155.6 (5)	C1B—N1B—C14—C15	84.4 (17)
C14—N3A—C13A—C8A	−29.7 (8)	C7B—N1B—C14—N3A	157 (2)
C11A—C12A—C13A—N3A	177.0 (6)	C1B—N1B—C14—N3A	−24.1 (15)
C11A—C12A—C13A—C8A	2.2 (9)	C7B—N1B—C14—N3B	17 (2)
C9A—C8A—C13A—N3A	−177.3 (6)	C1B—N1B—C14—N3B	−164.6 (17)
C7A—C8A—C13A—N3A	0.1 (9)	C13B—N3B—C14—N1A	−21.5 (15)
C9A—C8A—C13A—C12A	−2.4 (9)	C13B—N3B—C14—C15	86.8 (17)
C7A—C8A—C13A—C12A	175.0 (6)	C13B—N3B—C14—N3A	−102.9 (18)
C7B—N1B—C1B—C6B	1(3)	C13B—N3B—C14—N1B	−26.6 (18)
C14—N1B—C1B—C6B	−177.7 (16)	N1A—C14—C15—C16	170.5 (4)
C7B—N1B—C1B—C2B	−178 (3)	N3A—C14—C15—C16	−68.9 (6)
C14—N1B—C1B—C2B	3(4)	N1B—C14—C15—C16	−150.5 (6)
C6B—C1B—C2B—C3B	1(4)	N3B—C14—C15—C16	104.5 (7)
N1B—C1B—C2B—C3B	−180 (3)	N1A—C14—C15—C20	−11.2 (7)
C1B—C2B—C3B—C4B	2(4)	N3A—C14—C15—C20	109.3 (5)
C2B—C3B—C4B—C5B	−3(5)	N1B—C14—C15—C20	27.7 (8)
C3B—C4B—C5B—C6B	0(5)	N3B—C14—C15—C20	−77.3 (7)
C2B—C1B—C6B—N2B	−180 (2)	C20—C15—C16—O1	179.3 (4)
N1B—C1B—C6B—N2B	1(3)	C14—C15—C16—O1	−2.4 (6)
C2B—C1B—C6B—C5B	−4(4)	C20—C15—C16—C17	1.0 (7)
N1B—C1B—C6B—C5B	176.6 (18)	C14—C15—C16—C17	179.3 (4)
C7B—N2B—C6B—C1B	−3(3)	O1—C16—C17—C18	178.8 (4)
C7B—N2B—C6B—C5B	−178 (2)	C15—C16—C17—C18	−3.0 (7)
C4B—C5B—C6B—C1B	3(4)	C16—C17—C18—O2	−177.4 (4)
C4B—C5B—C6B—N2B	178 (2)	C16—C17—C18—C19	3.6 (7)
C6B—N2B—C7B—N1B	4(2)	O2—C18—C19—C20	178.7 (4)
C6B—N2B—C7B—C8B	179 (3)	C17—C18—C19—C20	−2.3 (7)
C1B—N1B—C7B—N2B	−3(3)	C18—C19—C20—C15	0.3 (7)
C14—N1B—C7B—N2B	175.5 (14)	C16—C15—C20—C19	0.4 (7)
C1B—N1B—C7B—C8B	−179 (2)	C14—C15—C20—C19	−177.9 (4)
C14—N1B—C7B—C8B	0(3)		

### Hydrogen-bond geometry (Å, °)

**Table:**

*D*—H···*A*	*D*—H	H···*A*	*D*···*A*	*D*—H···*A*
O1—H1O1···S1A	0.94	2.82	3.732 (3)	163
O1—H1O1···O3A	0.94	1.71	2.619 (9)	163
O2—H1O2···N2A^i^	0.88	1.95	2.739 (6)	150
C11A—H11A···O2^ii^	0.93	2.40	3.329 (9)	174

Symmetry codes: (i) *x*−1, *y*, *z*; (ii) −*x*+1, *y*−1/2, −*z*+1/2.
